# IMPPY3D: Image Processing in Python for 3D Image Stacks

**DOI:** 10.21105/joss.07405

**Published:** 2025

**Authors:** Newell H. Moser, Alexander K. Landauer, Orion L. Kafka

**Affiliations:** 1 Material Measurement Laboratory, National Institute of Standards and Technology, 325 Broadway, Boulder, CO, 80305, USA; 2 Material Measurement Laboratory, National Institute of Standards and Technology, 100 Bureau Drive, Gaithersburg, 20899, MD, USA

## Abstract

Image Processing in Python for 3D image stacks, or IMPPY3D, is a free and open-source software (FOSS) repository that simplifies post-processing and 3D shape characterization for grayscale image stacks, otherwise known as volumetric images, 3D images, or voxel models. While IMPPY3D, pronounced impee-three-dee, was originally created for post-processing image stacks generated from X-ray computed tomography (XCT) measurements, it can be applied generally in post-processing 2D and 3D images. IMPPY3D includes tools for segmenting volumetric images and characterizing the 3D shape of features or regions of interest. These functionalities have proven useful in 3D shape analysis of powder particles, porous polymers, concrete aggregates, internal pores/defects, and more (see the Research Applications section). IMPPY3D consists of a combination of original Python scripts, Cython extensions, and convenience wrappers for popular third-party libraries like SciKit-Image (Walt et al., 2014), OpenCV (Bradski, 2000), and PyVista (Sullivan & Kaszynski, 2019).

Highlighted capabilities of IMPPY3D include: varying image processing parameters interactively, applying numerous 2D/3D image filters (e.g., blurring/sharpening, denoising, erosion/dilation), segmenting and labeling continuous 3D objects, precisely rotating and re-slicing an image stack in 3D, generating rotated bounding boxes fitted to voxelized features, converting image stacks into 3D voxel models, exporting 3D models as Visualization Toolkik (VTK) files for ParaView (Ayachit, 2015), and converting voxel models into smooth mesh-based models. Additional information and example scripts can be found in the included ReadMe files within the IMPPY3D GitHub repository (Moser, Landauer, et al., 2024). As a visualized example, Figure 1 demonstrates the high-level steps to characterize powder particles using IMPPY3D. This workflow is also similar to how pores can be visualized and characterized in metal-based additive manufacturing. Additional research applications for IMPPY3D are discussed in a later section.

## Statement of Need

Volumetric images commonly arise from nondestructive measurement techniques such as XCT, optical coherence tomography (OCT) or confocal microscopy, or from destructive techniques such as serial sectioning. Volumetric images typically analyzed in IMPPY3D are grayscale representations of a 3D volume of material and contain both internal and external shape information. For example, XCT is commonly used in metal-based additive manufacturing to prevent parts from entering service that contain critical internal defects. Post-reconstruction image analysis software is often employed to post-process volumetric images, such as Dragonfly^1^ (Dragonfly 3D World, 2024) and Avizo (Avizo, 2024). While closed-source software packages are highly sophisticated tools, they are also inherently limited since users are restricted by the closed-source publishing model. Users may require specific features that are unavailable, and closed-source models can be difficult or impossible to validate and verify.

Non-commercial software packages are also available that post-process volumetric images with varying degrees of generality and openness. While not an exhaustive list, examples include ImageJ/FIJI (Schindelin et al., 2012), 3D Slicer (Kikinis et al., 2013), DREAM.3D (Groeber & Jackson, 2014), SPAM (Stamati et al., 2020), and PuMA (Ferguson et al., 2021). However, existing software can be difficult to extend for custom analyses, and/or their current features and strengths lie outside of volumetric segmentation and 3D shape characterization.

There are also FOSS packages that specialize in tomographic reconstruction, such as TomoPy (Gürsoy et al., 2014) and Tomviz (Tomviz, 2024). However, the focus of IMPPY3D is the segmentation and feature analysis of already-reconstructed 3D image stacks, rather than image reconstruction itself. IMPPY3D is written in straightforward Python that contains internal documentation with the goal of making the library flexible and extensible to anyone with basic knowledge of Python and image processing. The library has been designed to work within an Conda/Miniforge environment for either Windows or Linux machines.

## Research Applications of IMPPY3D

IMPPY3D has been in development since 2021. During this period, the library has evolved and been used in several research thrusts at the National Institute of Standards and Technology (NIST). Examples of published research applications, mostly related to XCT, include additive manufacturing, impact mitigating foams, powder particles, concrete aggregates, and lunar soil/regolith. A non-exhaustive list of publications involving IMPPY3D includes:

- Goguen et al. (2024), Three-dimensional characterization of particle size, shape, and internal porosity for Apollo 11 and Apollo 14 lunar regolith and JSC-1A lunar regolith soil simulant
- Moser, Benzing, et al. (2024), AM Bench 2022 Macroscale Tensile Challenge at Different Orientations (CHAL-AMB2022-04-MaTTO) and Summary of Predictions
- Kafka et al. (2024), A technique for in-situ displacement and strain measurement with laboratory-scale X-ray Computed Tomography
- Landauer, Kafka, et al. (2023), A materials data framework and dataset for elastomeric foam impact mitigating materials
- Landauer, Tsinas, et al. (2023), Unintended consequences: Assessing thermo-mechanical changes in vinyl nitrile foam due to micro-computed X-ray tomographic imaging
- Derimow et al. (2022), Surface globularization generated by standard PBF-EB Ti-6Al-4V processing achieves an improvement in fatigue performance

## Getting Started with IMPPY3D

To begin using IMPPY3D, a Python environment with the necessary dependencies must be installed. We have deployed the code using the open-source package manager “Mamba” from Miniforge (version 24.3.0) based on Python 3.10 (Miniforge, 2024). The IMPPY3D GitHub repository (Moser, Landauer, et al., 2024) contains a dependencies folder which provides environment files (.yml) and a “ReadMe.txt” file that explains how to install a new Python environment using these environment files. In addition to “Mamba” (or “Conda” for Anaconda users), there are also generic instructions on how to install the necessary dependencies using PIP. Currently, IMPPY3D has been tested to work on modern Windows and Linux machines for Python versions 3.9 and 3.10. For users to test the success of the installation of the Python environment, there are example scripts in the “examples” folder in the IMPPY3D GitHub repository. These examples are also documented in a “ReadMe.txt” file.

**Figure 2:**
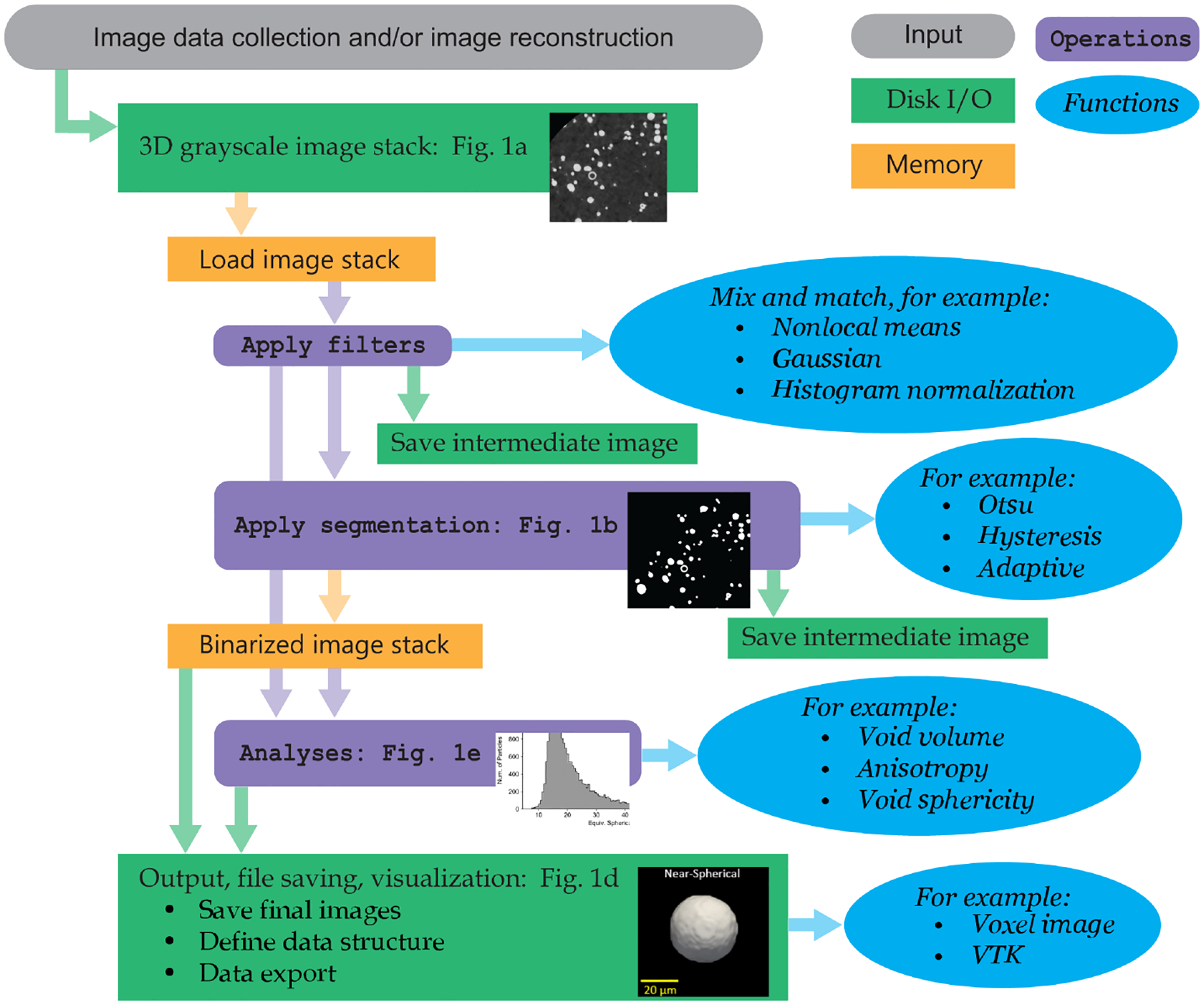
A high-level processing pipeline diagram illustrating typical steps and options available in IMPPY3D for 3D image stacks.

In summary, IMPPY3D is a library of tools designed to accelerate the post-processing of image stacks. The package does not include a graphical user-interface (GUI). Therefore, users are expected to write their own Python scripts that utilize the IMPPY3D library, and the provided examples serve as templates that illustrate how to use a wide range of the functionality available in IMPPY3D. Typical processing pipeline options in IMPPY3D is illustrated in Figure 2.

## Figures and Tables

**Figure 1:**
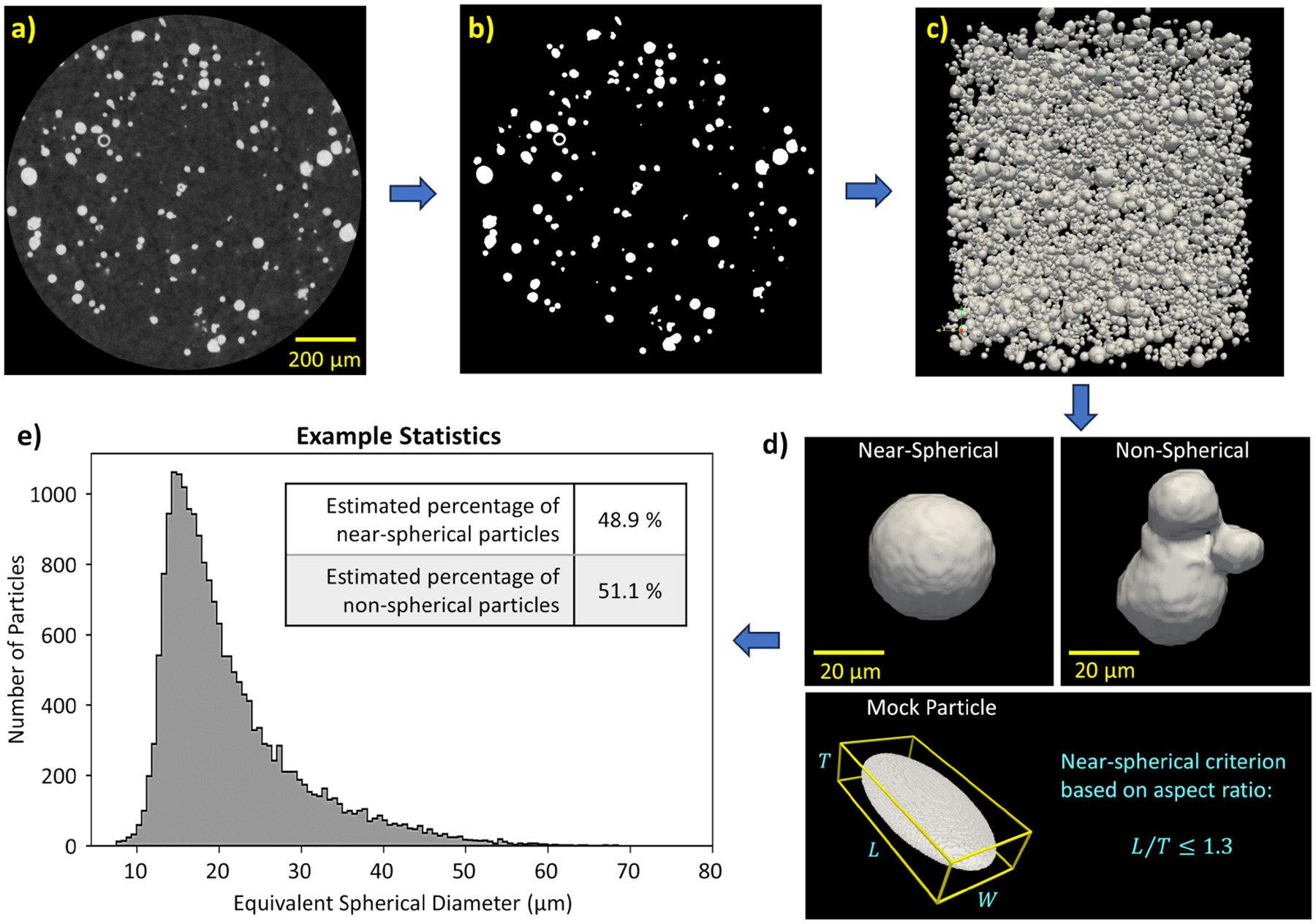
X-ray computed tomography reconstructions of nickel-based powder particles suspended in cured epoxy. a) One reconstructed 2D image slice (out of 1009) illustrating the powder particles, and b) the same image after segmentation using a series of filtering and binarization techniques. c) A rendering of the interactive 3D model of the segmented particle volume image. d) Individual particles visualized for characterization based on shape, volume, and porosity. e) The ratio of spherical to non-spherical particles and a histogram plot showing the distribution in size of the particles.

## Footnotes

1 Certain equipment, instruments, software, or materials are identified in this paper in order to specify the data processing procedure adequately. Such identification is not intended to imply recommendation or endorsement of any product or service by NIST, nor is it intended to imply that the materials or equipment identified are necessarily the best available for the purpose.
